# A Novel Immune-Related Prognostic Biomarker and Target Associated With Malignant Progression of Glioma

**DOI:** 10.3389/fonc.2021.643159

**Published:** 2021-04-16

**Authors:** Yu Zhang, Xin Yang, Xiao-Lin Zhu, Zhuang-Zhuang Wang, Hao Bai, Jun-Jie Zhang, Chun-Yan Hao, Hu-Bin Duan

**Affiliations:** ^1^ Department of Neurosurgery, First Hospital of Shanxi Medical University, Taiyuan, China; ^2^ Department of Geriatrics, First Hospital of Shanxi Medical University, Taiyuan, China; ^3^ Department of Neurosurgery, Lvliang People’s Hospital, Lvliang, China

**Keywords:** glioma, prognosis, immune, malignant progression, TNFRSF12A

## Abstract

**Background:**

Glioma is one of the most common malignancies in the central nervous system and has limited effective therapeutic options. Therefore, we sought to identify a suitable target for immunotherapy.

**Materials and Methods:**

We screened prognostic genes for glioma in the CGGA database and GSE43378 dataset using survival analysis, receiver operating characteristic (ROC) curves, independent prognostic analysis, and clinical correlation analysis. The results were intersected with immune genes from the ImmPort database through Venn diagrams to obtain likely target genes. The target genes were validated as prognostically relevant immune genes for glioma using survival, ROC curve, independent prognostic, and clinical correlation analyses in samples from the CGGA database and GSE43378 dataset, respectively. We also constructed a nomogram using statistically significant glioma prognostic factors in the CGGA samples and verified their sensitivity and specificity with ROC curves. The functions, pathways, and co-expression-related genes for the glioma target genes were assessed using PPI networks, enrichment analysis, and correlation analysis. The correlation between target gene expression and immune cell infiltration in glioma and the relationship with the survival of glioma patients were investigated using the TIMER database. Finally, target gene expression in normal brain, low-grade glioma, and high-grade glioma tissues was detected using immunohistochemical staining.

**Results:**

We identified TNFRSF12A as the target gene. Satisfactory results from survival, ROC curve, independent prognosis, and clinical correlation analyses in the CGGA and GSE43378 samples verified that TNFRSF12A was significantly associated with the prognosis of glioma patients. A nomogram was constructed using glioma prognostic correlates, including TNFRSF12A expression, primary-recurrent-secondary (PRS) type, grade, age, chemotherapy, IDH mutation, and 1p19q co-deletion in CGGA samples with an AUC value of 0.860, which illustrated the accuracy of the prognosis prediction. The results of the TIMER analysis validated the significant correlation of TNFRSF12A with immune cell infiltration and glioma survival. The immunohistochemical staining results verified the progressive up-regulation of TNFRSF12A expression in normal brain, low-grade glioma, and high-grade glioma tissues.

**Conclusion:**

We concluded that TNFRSF12A was a viable prognostic biomarker and a potential immunotherapeutic target for glioma.

## Introduction

Gliomas are the most common primary intracranial tumors, accounting for 81% of intracranial malignancies (1). In the past, the categories of astrocytoma, oligodendroglioma, oligoastrocytoma, and ependymoma, were commonly used for the pathological classification of gliomas in clinical practice (2). The World Health Organization (WHO) recently proposed a novel glioma classification method based on the presence or absence of IDH mutations and 1p/19q co-deletion (3). These new classifications have effectively promoted the progress of molecular diagnosis and treatment of glioma, resulting in molecular detection becoming an increasingly important component of glioma diagnosis and treatment. With rapid developments in biomedical research, the procedures for tumor exploration, localization, and surgical treatment of brain tumors are gradually improving. However, conventional surgical resection cannot completely remove all brain tumors, and often, residual tumor cells remain. Although postoperative chemoradiotherapy can prolong the survival of some patients, the overall prognosis is poor (1, 4–7). Suppression or eradication of glioma cells through specific immune targets is a potential therapeutic strategy to improve glioma treatment. For example, it has been reported that the accumulation of regulatory T (T reg) cells in glioblastoma (GBM) contributes to the suppression of anti-tumor immunity, and the combined blockade of IL-12 and CTLA-4 acts on CD4 (+) cells, resulting in the reduction of FoxP3 (+) T reg cells and increases in effector T cells, thereby inhibiting tumor growth (8). Also, evidence suggests that elevated expression of PD-L1 protein can suppress immune processes (9). PD-L1 inhibition therapy resulted in a significant inhibitory effect on GBM (10). Thus, to identify similar novel immune-related prognostic markers and targets, we used CGGA, GEO, and ImmPort databases to screen for glioma prognostication-related immune genes. We analyzed and validated the possibility that the identified genes could serve as glioma prognostic markers and therapeutic targets using various methods.

## Materials and Methods

### Data Preparation

The mRNAseq 693 and mRNAseq 325 glioma sample data were downloaded from the Chinese Cerebral Glioma Genome Atlas (CGGA) database (http://www.cgga.org.cn/download.jsp), which contains mRNA expression and clinical profiles, for which samples with incomplete information had been previously identified and separated out. The GSE43378 chip-containing gene expression profiles and clinical profiles for the glioma samples were extracted from the Gene Expression Omnibus (GEO) database (https://www.ncbi.nlm.nih.gov/geo/). The GSE43378 profile was based on the GPL570 [HG-U133 Plus2] Affymetrix Human Genome U133 Plus 2.0 Array platform. We performed gene annotation for the mRNA expression data from the glioma samples in the CGGA database and GSE43378 data set, respectively. The expression was processed by taking log2 [data = log2(data+1)] and correcting for the batch effect, then combined with the clinical data.

### Target Gene Screening

**Figure 1:**
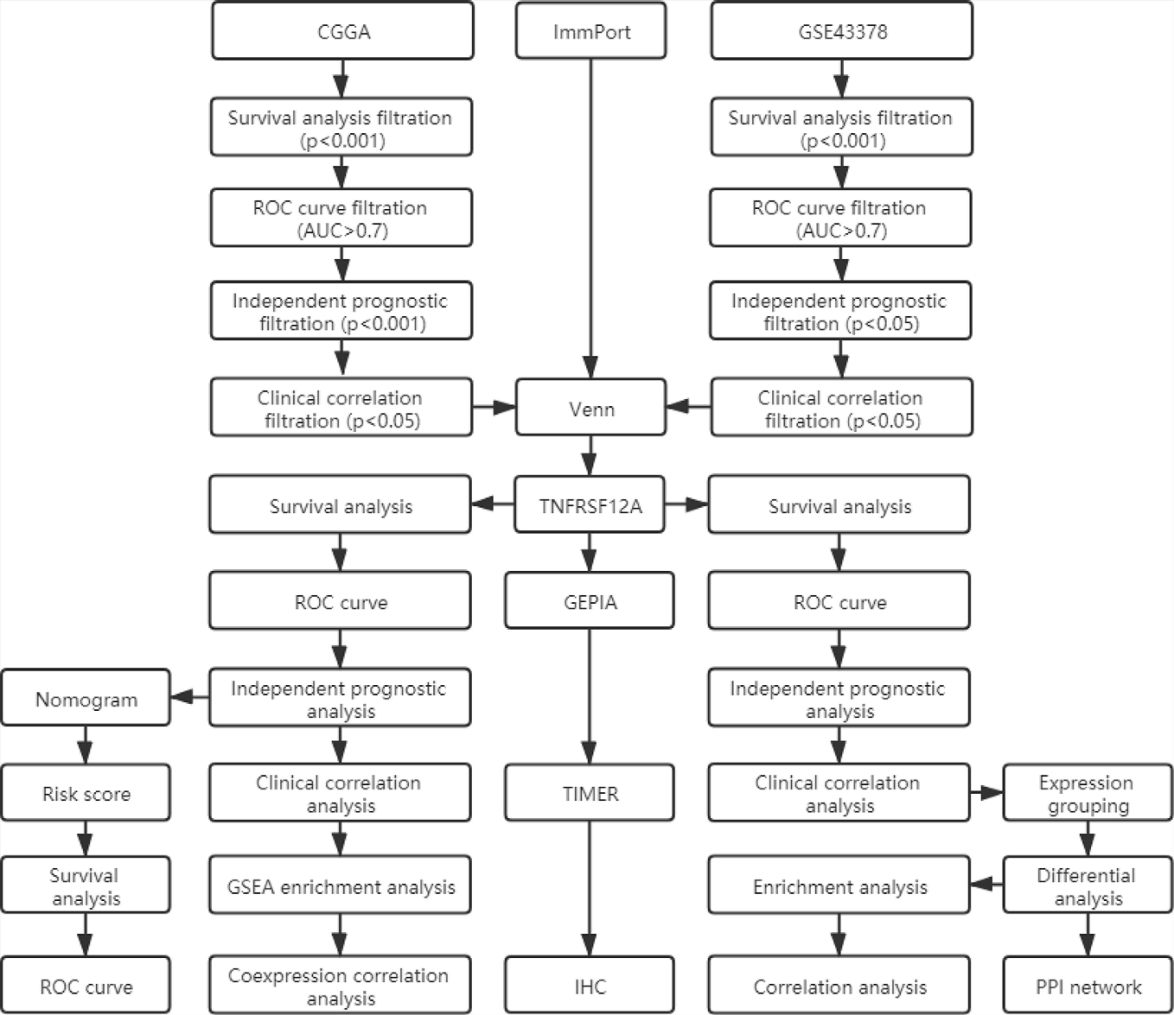
The work flow diagram of this study.

We obtained a list of immune-related genes from the ImmPort database (https://www.immport.org/shared/home). The ImmPort project is a platform for collecting, organizing, and sharing immunology-related research. The platform contained experimental data and metadata that described the study objectives and data generation methods. We conducted survival, ROC curve, independent prognostic, and clinical correlation analyses to batch screen the genes from the CGGA and GSE43378 glioma samples. We selected the gene clusters that were significantly associated with the survival, prognosis, and clinical features of glioma patients. The filtration criteria are shown in **Figure 1**. A p-value <0.05 was set as the threshold for statistically significant differences. The intersection was determined between glioma-related genes and ImmPort immune-related genes using the Venn online web program (http://bioinformatics.psb.ugent.be/webtools/Venn/). The less studied single genes that were identified were selected for additional analysis. Single gene expression data were extracted and combined with clinical data. The differential expression of target genes in low-grade glioma (LGG), GBM, and normal samples was verified using the GEPIA database (http://gepia.cancer-pku.cn/), which integrated gene expression profiles from the TCGA and GTEx projects and contained RNA sequencing expression profiles from 9,736 tumors and 8,587 normal samples.

### Survival Analysis

We used R statistical software (Version 4.0.2) to conduct all survival analyses. The samples from the CGGA and GSE43378 data were initially grouped based on median single gene expression. Survival analysis was conducted in the different groups to detect the correlation between gene expression levels and glioma prognosis in the patients. Differences of p < 0.05 were considered to be statistically significant.

### Receiver Operating Characteristic (ROC) Curves

To validate the accuracy of single genes for predicting survival in patients with glioma, 1-year, 3-year, and 5-year survival ROC curves were plotted using R statistical software (Version 4.0.2). Area under curve (AUC) values were calculated to assess the validity of the model. AUC values of 0.5–0.7 were considered moderate, 0.7–0.9 were considered better, and >0.9 was superior.

### Independent Prognostic Analysis

To determine the prognostic factors associated with glioma patients, we performed univariate and multivariate independent prognostic analyses on the samples from the CGGA and GSE43378 data using R statistical software (Version 4.0.2). The variables used for screening the CGGA data included single gene expression, primary-recurrent-secondary (PRS) type, histology, grade, gender, age, radiation therapy, chemotherapy, IDH mutations, and 1p19q co-deletion. The variables used to screen the GSE43378 data included single gene expression, histology, grade, gender, and age. Age was stratified at 41 years. Differences of p < 0.05 were considered statistically significant. Non-statistically significant variables were excluded.

### Prognostic Nomogram Construction

A nomogram was constructed based on statistically significant prognostic factors collected from the CGGA data using R statistical software (Version 4.0.2), and risk scores were calculated. Based on the median risk score value, samples were classified into high- and low-risk groups. Survival analysis was performed for the different groups. A p-value <0.05 was considered to be statistically significant. We used ROC curves to assess the sensitivity and specificity of the nomogram model.

### Clinical Correlation Analysis

To evaluate the correlation between single genes and clinical features of glioma patients, we performed correlation analysis for single gene expression and clinical features for the CGGA and GSE43378 data, respectively. A p-value <0.05 indicated that single genes were significantly correlated with the corresponding clinical features.

### Differential Analysis, PPI Network, and Enrichment Analysis

The glioma samples from the GSE43378 dataset were grouped based on median single gene expression. Differentially expressed genes (DEGs) were obtained through analysis (|logFC|> 0.5, adjP < 0.05), and volcano plots were constructed. Twenty of the highest significantly up-regulated and 20 of the lowest significantly down-regulated DEGs were extracted to plot correlation heat maps. Potential protein interactions between the DEGs were assessed using the STRING database (https://string-db.org/). Based on a score of >0.4 as the PPI extraction criterion, a PPI network was visualized using Cytoscape software (www.cytoscape.org/). Gene Ontology (GO) function annotation and KEGG pathway enrichment analysis were conducted to explore the functions and pathways enriched with the DEGs (Count ≥ 10, adjP < 0.05). We also grouped the samples in the CGGA database using median single gene expression values. GSEA enrichment analysis was performed for the different groups. Finally, we obtained the top five GO function and KEGG pathway enriched by sample genes in high-expression group and low-expression group (p < 0.05).

### Correlation Analysis

We analyzed the correlation between the target genes and the sample genes from the CGGA and GSE43378 data and selected the genes that were significantly correlated with the target (cor > 0.5, p < 0.001). The correlation analysis was conducted using the limma package from R statistical software (Version 4.0.2). The top 20 genes in the CGGA and GSE43378 data that were positively correlated or negatively correlated with the target gene were selected and used to construct a correlation heat map. Finally, the top five genes in the CGGA and GSE43378 data that were positively and negatively correlated with the target genes were selected and plotted as correlation circles.

### Immune Cell Infiltration

The TIMER database (https://cistrome.shinyapps.io/timer/) was used to detect the infiltration of immune cells in tumor tissues based on RNA-Seq expression. Six types of immune cells were assessed, including B cells, CD4+ T cells, CD8+ T cells, neutrophils, macrophages, and dendritic cells. The gene module for TIMER was used to assess the correlation between single gene expression levels and infiltration of the six immune cell types in LGG and GBM patients. The survival module was used to assess the correlation between single genes, the six types of immune cell infiltration, and survival of LGG and GBM patients using the Cox proportional risk model. TIMER also was used to draw Kaplan-Meier plots for immune infiltration and genes to visualize differences in survival. The split percentage of patients (% = 50%) with a p-value <0.05 was considered to be statistically significant.

### Immunohistochemistry

Immunohistochemical staining was used to detect the expression of target immune-related genes in normal brain, low-grade glioma, and high-grade glioma tissues. The experiments that utilized human tissue were approved by the ethics committee of the First Hospital of Shanxi Medical University. Two samples of normal brain tissue in patients with epilepsy, two samples of low-grade glioma, and four high-grade glioma samples were collected from the First Hospital of Shanxi Medical University. All postoperative tissues were examined pathologically in the Department of Pathology, First Hospital of Shanxi Medical University. After routine paraffin-embedding, tissue sections were obtained, placed on glass microscope slides, de-paraffinized, and rehydrated. Antigen retrieval and blocking of endogenous peroxidases were performed, followed by exposure to monogenic polyclonal antibodies (Sangon, Shanghai, China) and enzyme-labeled IgG polymers. Antibody presence was visualized using a diaminobenzidine (DAB) chromogenic solution and hematoxylin as a counterstain.

## Results

### Target Gene Identification

**Figure 2:**
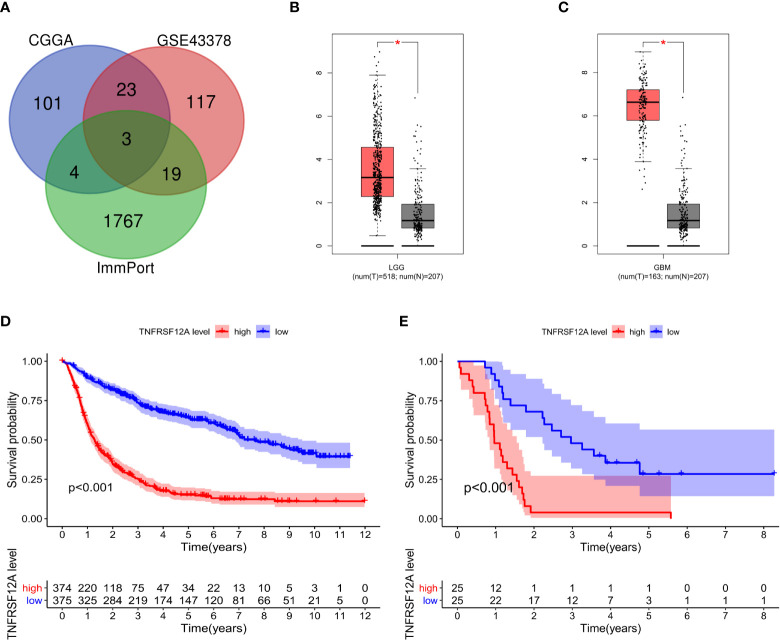
**(A)** Venn diagram identifying the intersection of glioma prognostic genes from CGGA and GSE43378 data and immune genes from ImmPort. **(B)** Expression of TNFRSF12A in LGG and normal samples. **(C)** Expression of TNFRSF12A in GBM and normal samples. **(D)** Survival analysis of glioma patients in the high and low expression groups of TNFRSF12A in CGGA. **(E)** Survival analysis of glioma patients in the high and low expression groups of TNFRSF12A in GSE43378. *p < 0.05

In this study, 131 glioma prognosis-associated genes were collected from CGGA samples through survival, ROC curve, independent prognostic, and clinical correlation analyses. In addition, 162 glioma prognosis-associated genes were collected from GSE43378 samples, and 1,793 immune-related genes were identified in the ImmPort immune gene list **(** **Table 1** **)**. The intersection of the glioma prognosis-related genes from the CGGA, GES43378, and immune-related genes from the ImmPort platform was identified using the Venn online web program **(** **Figure 1** **)**. Three immune genes related to glioma prognosis were identified as TGFB2, VIM, and TNFRSF12A **(** **Figure 2A** **)**. We chose TNFRSF12A as the target of our study. The results of the GEPIA analysis revealed that the expression of TNFRSF12A was significantly up-regulated in LGG and GBM patient tissues compared to normal tissues **(** **Figures 2B, C** **)**.

**Table 1:** The number of samples and genes screened in CGGA and GSE43378.

Database ID	Sample	Gene
CGGA	749	131
GSE43378	50	162
ImmPort	–	1,793
Total number	799	2,086

### Survival Analysis and ROC Curves

**Figure 3:**
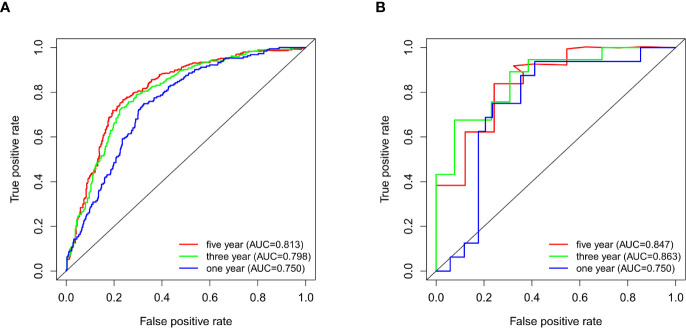
**(A)** ROC curves for 1-, 3-, and 5-year survival of patients in CGGA. **(B)** ROC curves for 1-, 3-, and 5-year survival of patients in GSE43378.

The survival data from glioma patients in the CGGA and GSE43378 datasets were significantly different between the high and low TNFRSF12A expression groups (p < 0.001). The survival time for the glioma patients in the high expression group was significantly shorter, suggesting that TNFRSF12A hyperexpression might be a risk factor for a poor prognosis of glioma patients **(** **Figures 2D, E** **)**. The AUC values for the 1-year, 3-year, and 5-year survival ROC curves for the CGGA samples were 0.813, 0.798, and 0.750, respectively **(** **Figure 3A** **)**, and for the GSE43378 samples were 0.847, 0.863, and 0.750, respectively **(** **Figure 3B** **)**. The AUC values for both groups were high, which validated the accuracy of TNFRSF12A as a prognostic gene in predicting survival time in glioma patients.

### Univariate and Multivariate Independent Prognostic Analyses

**Figure 4:**
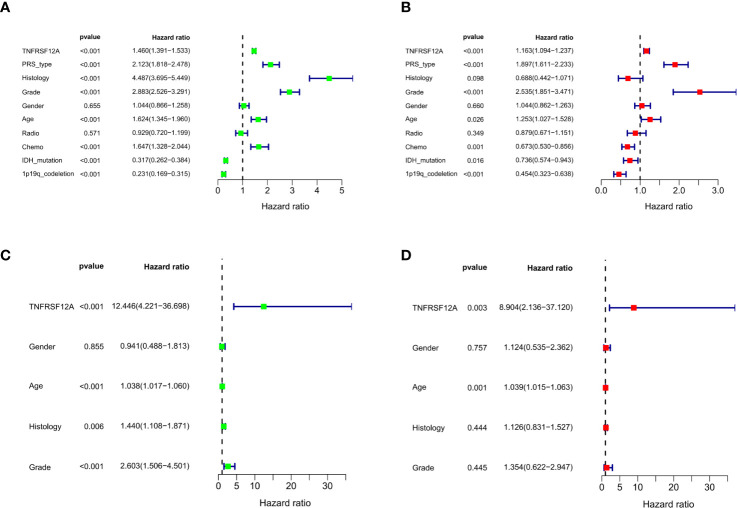
**(A)** Univariate independent prognostic analysis of samples in CGGA. **(B)** Multivariate independent prognostic analysis of samples in CGGA. **(C)** Univariate independent prognostic analysis of samples in GSE43378. **(D)** Multivariate independent prognostic analysis of samples in GSE43378.

Three variables unrelated to glioma prognosis were excluded by univariate and multivariate independent prognostic analysis in the CGGA data, which were histology, gender, and radiation therapy (p > 0.05, **Figures 4A, B** **)**. Three variables were excluded in the GSE43378 data, which were histology, grade, and gender (p > 0.05, **Figures 4C, D** **)**. Independent prognostic analysis of both groups illustrated that the expression level of TNFRSF12A was an independent prognostic factor for glioma **(** **Table 2** **)**.

**Table 2:** Univariate and multivariate independent prognostic analysis of glioma patients in CGGA and GSE43378.

	Parameters	Univariate analysis				Multivariate analysis				
		HR	HR.95L	HR.95H	p value	HR	HR.95L	HR.95H	p value	
CGGA	TNFRSF12A	1.46	1.39	1.53	5.41E-53	1.16	1.09	1.23	1.37E-06	<0.05
	PRS_type	2.12	1.81	2.47	1.79E-21	1.89	1.61	2.23	1.47E-14	<0.05
	Histology	4.48	3.69	5.44	7.38E-52	0.68	0.44	1.07	0.097813569	
	Grade	2.88	2.52	3.29	1.44E-55	2.53	1.85	3.47	6.67E-09	<0.05
	Gender	1.04	0.86	1.25	0.655307114	1.04	0.86	1.26	0.659748289	
	Age	1.62	1.34	1.96	4.49E-07	1.25	1.02	1.52	0.026094249	<0.05
	Radio	0.92	0.71	1.19	0.570623486	0.87	0.67	1.15	0.349417608	
	Chemo	1.64	1.32	2.04	5.71E-06	0.67	0.52	0.85	0.001260157	<0.05
	IDH_mutation	0.31	0.26	0.38	3.84E-32	0.73	0.57	0.94	0.015520671	<0.05
	1p19q_codeletion	0.23	0.16	0.31	2.08E-20	0.45	0.32	0.63	5.19E-06	<0.05
GSE43378	TNFRSF12A	12.44	4.22	36.69	4.87E-06	8.90	2.13	37.11	0.00268473	<0.05
	Gender	0.94	0.48	1.81	0.855389498	1.12	0.53	2.36	0.757094847	
	Age	1.03	1.01	1.06	0.000468367	1.03	1.01	1.06	0.001305705	<0.05
	Histology	1.43	1.10	1.87	0.006370135	1.12	0.83	1.52	0.44409736	
	Grade	2.60	1.50	4.50	0.000616173	1.35	0.62	2.94	0.444637821	

### Prognostic Nomogram Construction

**Figure 5:**
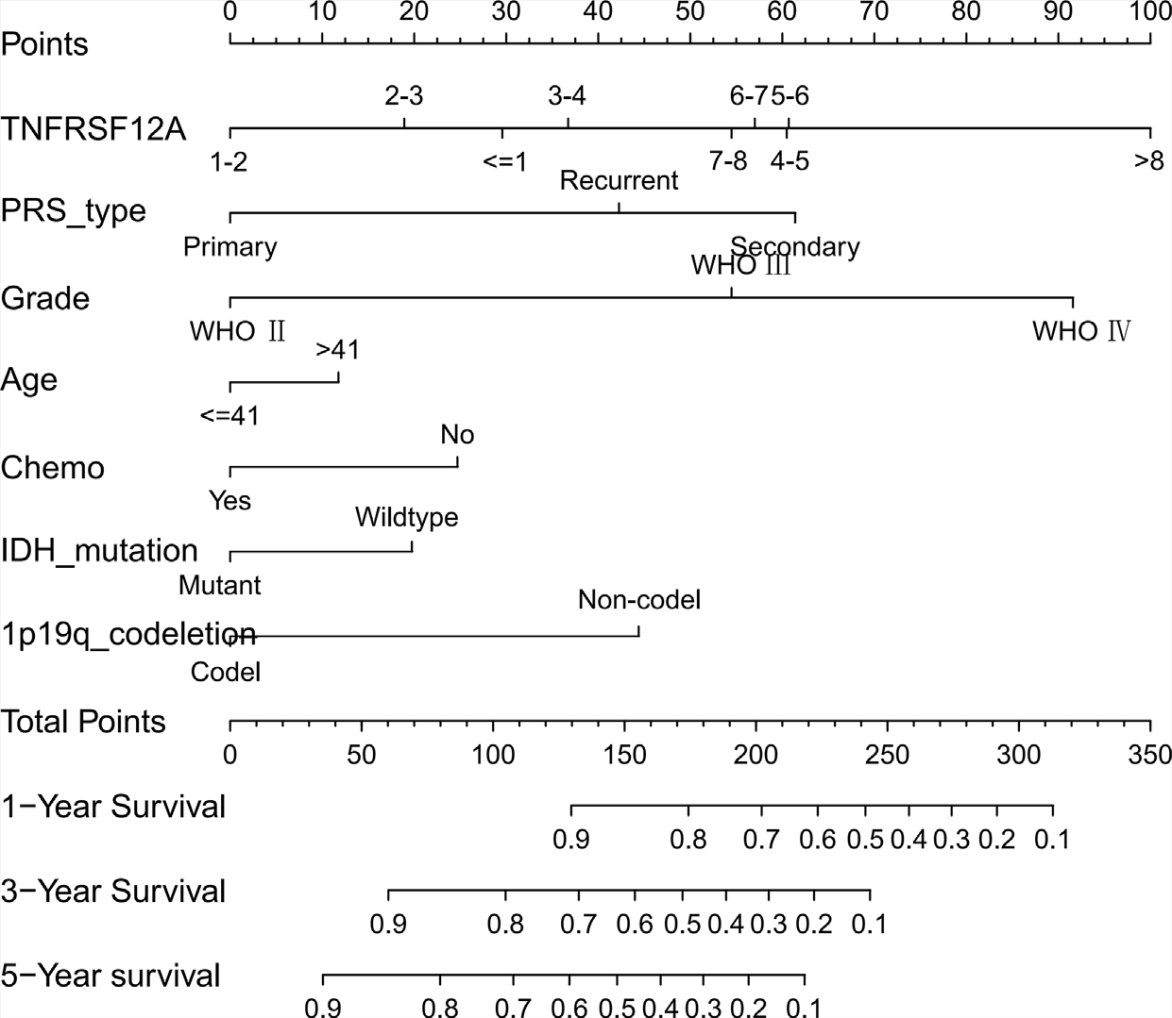
Nomogram of 1-year, 3-year, and 5-year survival in glioma patients with CGGA.

**Figure 6:**
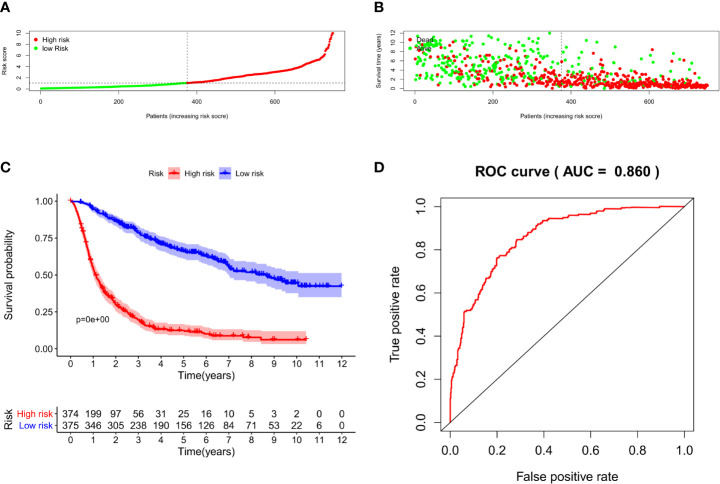
**(A)** Risk score grouping of samples in CGGA. **(B)** Survival time corresponding to the patient risk score in CGGA. **(C)** Survival analysis of patients in high-risk and low-risk groups. **(D)** ROC curve for the nomogram.

We constructed a nomogram based on seven meaningful prognosis-related variables in the CGGA data, including TNFRSF12A expression, PRS type, grade, age, chemotherapy, IDH mutations, and 1p19q co-deletion. One-year, 3-year, and 5-year survival rates for glioma patients were predicted based on the nomogram score **(** **Figure 5** **)**. The results of the risk curve and survival analyses revealed that low-risk patients survived longer than high-risk patients **(** **Figures 6A–C** **)**. The AUC value of the ROC curve was 0.860, which validated the accuracy of the nomogram **(** **Figure 6D** **)**.

### Clinical Correlation Verification

**Figure 7:**
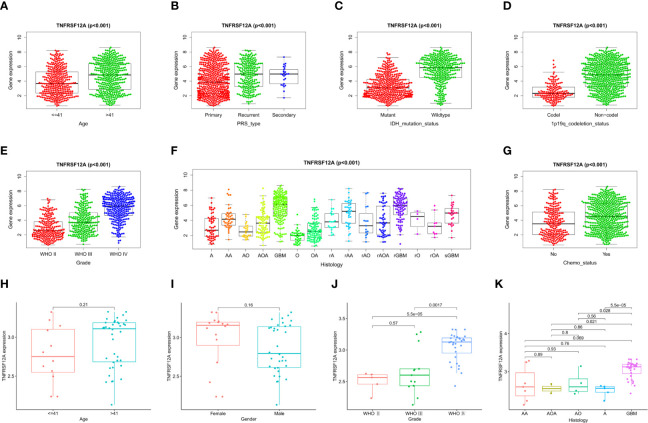
Correlation analysis of TNFRSF12A expression levels and clinical features in glioma patients. CGGA: **(A)** Age. **(B)** PRS type. **(C)** IDH mutation. **(D)** 1p19q co-deletion. **(E)** Grade. **(F)** Histology. **(G)** Chemotherapy. GSE43378: **(H)** Age, **(I)** Gender, **(J)** Grade. **(K)** Histology.

Clinical correlation analysis of the CGGA data demonstrated that the expression level for TNFRSF12A was significantly correlated with PRS type, histology, grade, age, chemotherapy, IDH mutations, and 1p19q co-deletion in glioma samples. TNFRSF12A expression was higher in patients >41 years of age compared to patients ≤41 years of age **(** **Figure 7A** **)** and higher in recurrent and secondary gliomas than in primary gliomas **(** **Figure 7B** **)**. TNFRSF12A exhibited higher expression in wildtype and 1p19q non-coding glioma compared to IDH mutants or 1p19q co-deletions **(** **Figures 7C, D** **)**. The expression of TNFRSF12A was up-regulated as the glioma grade increased **(** **Figure 7E** **)**. GBM patients exhibited the highest expression levels among all subtypes **(** **Figure 7F** **)**. TNFRSF12A expression decreased in patients who underwent chemotherapy **(** **Figure G** **)**. Expression of TNFRSF12A was significantly correlated with histology and grade in glioma patients and independent of age and gender, as seen in the GSE43378 samples **(** **Figures 7H, I** **)**. Moreover, TNFRSF12A expression was up-regulated as the glioma grade increased **(** **Figure 7J** **).** The expression of TNFRSF12A in GBM patients was highest among all glioma subtypes **(** **Figure 7K** **)**.

### Differential Analysis, PPI Network, and Enrichment Analysis

**Figure 8:**
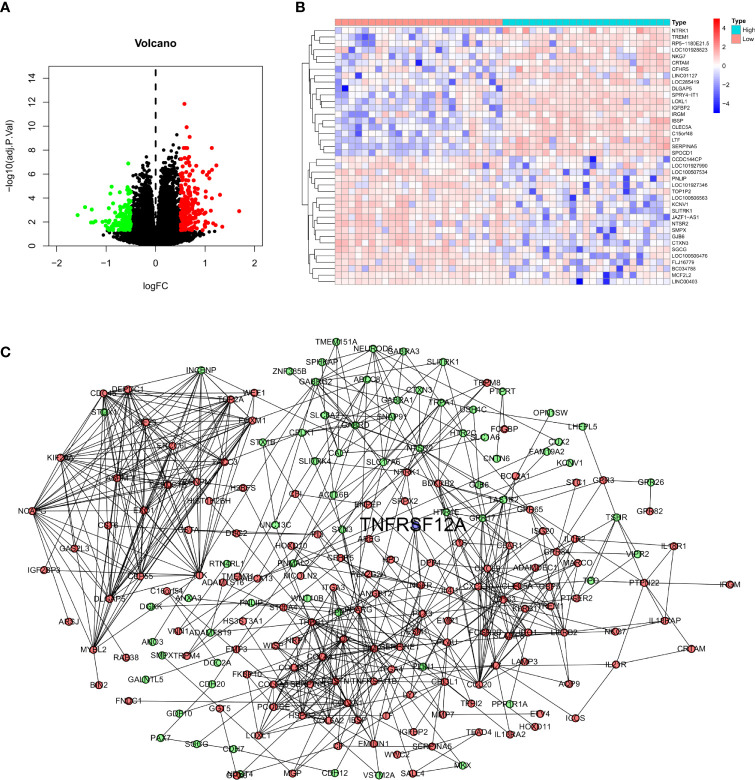
**(A)** Volcano map of DEGs in GSE43378. **(B)** Correlation heat map of the 20 up- and down-regulated most significant DEGs in GSE43378. **(C)** PPI network for DEGs of GSE43378 in String database, red dots indicate up-regulated genes, green dots indicate down-regulated genes, and blue dots indicate target gene.

**Figure 9:**
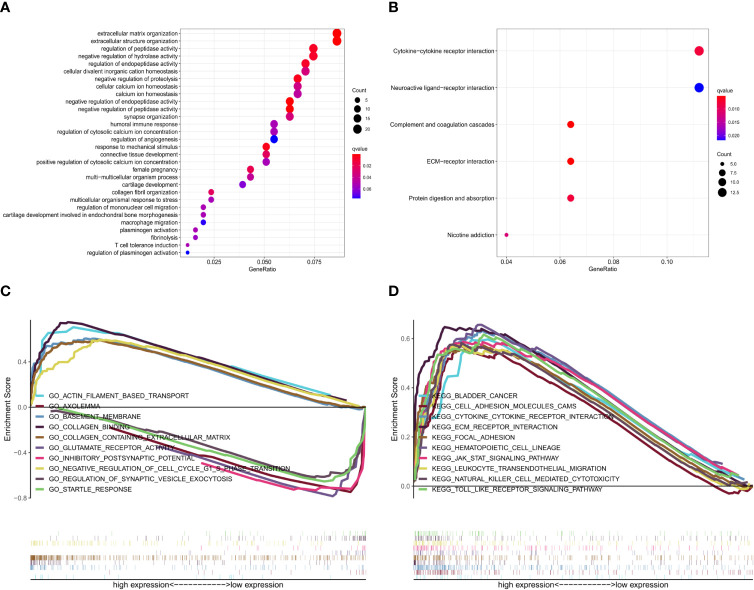
**(A)** GO enrichment analysis of DEGs in GSE43378. **(B)** KEGG pathway enrichment analysis of DEGs in GSE43378. **(C)** GSEA GO enrichment analysis of genes in CGGA. **(D)** GSEA KEGG pathway enrichment analysis of genes in CGGA.

Glioma samples from GSE43378 samples were grouped based on the median expression level of TNFRSF12A for differential analysis, and 645 DEGs were identified **(** **Figure 8A** **)**. Twenty of the highest significantly up-regulated and 20 of the lowest significantly down-regulated DEGs were plotted using a heat map **(** **Figure 8B** **)**. Potential protein interactions between the DEGs were assessed using the STRING online database. Two hundred three nodes and 642 edges of the PPI network were visualized using Cytoscape software **(** **Figure 8C** **)**. The enrichment analysis for the DEGs revealed that the main enriched GO functions of the DEGs were extracellular matrix organization, extracellular structure organization, regulation of peptidase activity, negative regulation of hydrolase activity, regulation of endopeptidase activity, negative regulation of proteolysis, negative regulation of endopeptidase activity, and negative regulation of peptidase activity **(** **Figure 9A** **)**. The DEGs were predominantly enriched for the cytokine-cytokine receptor interaction and neuroactive ligand-receptor interaction pathways **(** **Figure 9B** **)**. The main enrichment GO functions for the sample genes from the TNFRSF12A high expression group in the CGGA data were actin filament-based transport, basement membrane, collagen binding, collagen-containing extracellular matrix, and negative regulation of cell cycle G1-S phase transition. The primary enriched GO functions for the low expression group were axolemma, glutamate receptor activity, inhibitory postsynaptic potential, and synaptic vesicle exocytosis regulation **(** **Figure 9C** **)**. The main enrichment pathways for the sample genes from the TNFRSF12A high expression group were bladder cancer, cell adhesion molecule cams, cytokine-cytokine receptor interaction, ECM receptor interaction, focal adhesion, hematopoietic cell lineage, the JAK/STAT signaling pathway, leukocyte transendothelial migration, natural killer cell-mediated cytotoxicity, and the Toll-like-receptor signaling pathway. No significantly enriched pathways were found in the low-expression group **(** **Figure 9D** **)**.

### Correlation Analysis

**Figure 10:**
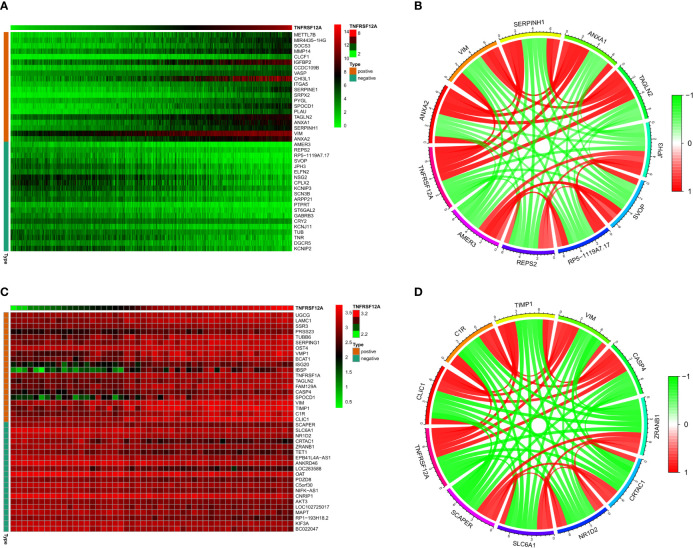
**(A)** Correlation heat map of the top 20 positively and negatively correlated genes with TNFRSF12A in CGGA. **(B)** Correlation circles for the top 5 positively and negatively correlated genes with TNFRSF12A in CGGA. **(C)** Correlation heat map of the top 20 positively and negatively correlated genes with TNFRSF12A in GSE43378. **(D)** Correlation circles for the top 5 positively and negatively correlated genes with TNFRSF12A in GSE43378.

The correlation heat map indicated that the 20 genes with the highest significant positive correlations with TNFRSF12A in the CGGA samples were ANXA2, VIM, SERPINH1, ANXA1, TAGLN2, PLAU, SPOCD1, PYGL, SRPX2, SERPINE1, ITGA5, CHI3L1, VASP, CCDC109B, IGFBP2, CLCF1, MMP14, SOCS3, MIR4435-1HG, and METTL7B. The 20 genes with the highest significant negative correlations were AMER3, REPS2, RP5-1119A7.17, SVOP, JPH3, ELFN2, NSG2, CPLX2, KCNIP3, SCN3B, ARPP21, PTPRT, ST6GAL2, GABRB3, CRY2, KCNJ11, TUB, TNR, DGCR5, and KCNIP2. The correlation diagram is seen in **Figure 10A**. The correlation circle shows the top five genes with positive and negative correlations **(** **Figure 10B** **)**. The correlation heat map showed that the 20 genes with the highest significant positive correlations with TNFRSF12A in the GSE43378 samples were CLIC1, C1R, TIMP1, VIM, SPOCD1, CASP4, FAM129A, TAGLN2, TNFRSF1A, IBSP, ISG20, BCAT1, VMP1, OST4, SERPING1, TUBB6, PRSS23, SSR3, LAMC1, and UGCG. The 20 genes with the highest significant negative correlations were SCAPER, SLC6A1, NR1D2, CRTAC1, ZRANB1, TET1, EPB41L4A-AS1, ANKRD46, LOC283588, OAT, PDZD8, C5orf30, NIFK-AS1, CNRIP1, AKT3, LOC102725017, MAPT, RP1-193H18.2, KIF3A, and BC022047 **(** **Figure 10C** **)**. The correlation circle shows the top five genes with positive and negative correlations **(** **Figure 10D** **)**.

### Immune Cell Infiltration

**Figure 11:**
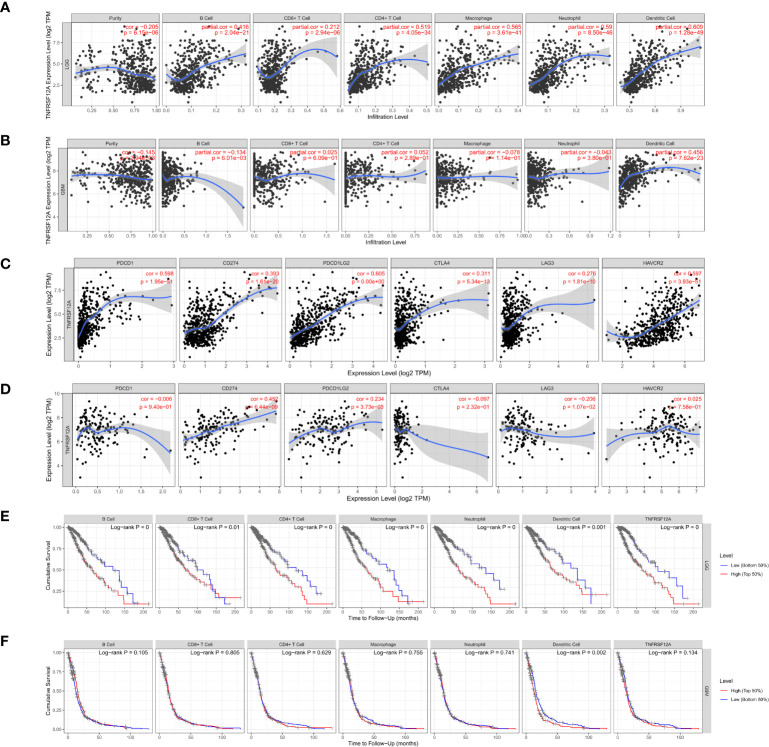
**(A)** Correlation between TNFRSF12A expression in LGG and infiltration of six immune cells. **(B)** Correlation between TNFRSF12A expression in GBM and infiltration of six immune cells. **(C)** Correlation between TNFRSF12A and six classical immune checkpoints in LGG. **(D)** Correlation between TNFRSF12A and six classical immune checkpoints in GBM. **(E)** KM curve of LGG patient survival with TNFRSF12A expression and infiltration of six immune cells. **(F)** KM curve of GBM patient survival with TNFRSF12A expression and infiltration of six immune cells.

Gene module analysis using TIMER demonstrated that TNFRSF12A expression in LGG patients was directly correlated with the infiltration of B cells, CD4+ T cells, CD8+ T cells, neutrophils, macrophages, and dendritic cells (all p < 0.05, **Figure 11A**). The expression of TNFRSF12A in GBM patients was inversely correlated with the infiltration of B cells (cor = −0.134, p = 6.01e-03), and directly correlated with the infiltration of dendritic cells (cor = 0.456, p = 7.62e-23) **(** **Figure 11B** **)**. TNFRSF12A expression in LGG patients showed a significant positive correlation with several classical immune checkpoints (PDCD1, CD274, PDCD1LG2, CTLA4, LAG3, and HAVCR2) (all p < 0.05, **Figure 11C**). TNFRSF12A expression in GBM patients showed a positive correlation with two classical immune checkpoints CD274 (cor = 0.452, p = 6.44e-09) and PDCD1LG2 (cor = 0.234, p = 3.73e-03), while LAG3 (cor = −0.206, p = 1.07e-02) exhibited a negative correlation **(** **Figure 11D** **)**. Analysis of Cox proportional risk models using the survival module showed that the infiltration of CD8+ T cells, macrophages, neutrophils, and the expression of TNFRSF12A were highly associated with the survival of LGG patients. Dendritic cell infiltration and TNFRSF12A expression were significantly associated with the survival of GBM patients **(** **Table 3** **)**. The Kaplan-Meier diagram showed that survival of LGG patients was significantly associated with the expression of TNFRSF12A and the infiltration of six types of immune cells, including B cells, CD4+ T cells, CD8+ T cells, neutrophils, macrophages, and dendritic cells (all p < 0.05, **Figure 11E**). The survival of GBM patients was correlated with the infiltration of dendritic cells (p = 0.002, **Figure 11F** **)**.

**Table 3:** Cox proportional risk model of TNFRSF12A expression and infiltration of six immune cells in LGG and GBM.

		coef	HR	95%CI_l	95%CI_u	p.value	sig
LGG	B_cell	2.741	15.499	0.052	4580.118	0.345	
	CD8_Tcell	7.710	2231.271	2.152	2312937.870	0.030	*
	CD4_Tcell	1.471	4.354	0.001	17479.969	0.728	
	Macrophage	4.311	74.512	1.262	4398.821	0.038	*
	Neutrophil	−8.154	0.000	0.000	0.598	0.036	*
	Dendritic	−1.202	0.301	0.005	17.319	0.561	
	TNFRSF12A	0.479	1.614	1.397	1.864	0.000	***
GBM	B_cell	−0.519	0.595	0.341	1.039	0.068	·
	CD8_Tcell	0.241	1.272	0.861	1.879	0.227	
	CD4_Tcell	0.135	1.144	0.600	2.182	0.683	
	Macrophage	0.069	1.071	0.573	2.003	0.830	
	Neutrophil	0.396	1.486	0.670	3.299	0.330	
	Dendritic	0.284	1.329	1.004	1.758	0.047	*
	TNFRSF12A	0.123	1.131	1.017	1.259	0.023	*

*P < 0.05, ***P < 0.001.

### Immunohistochemistry

**Figure 12:**
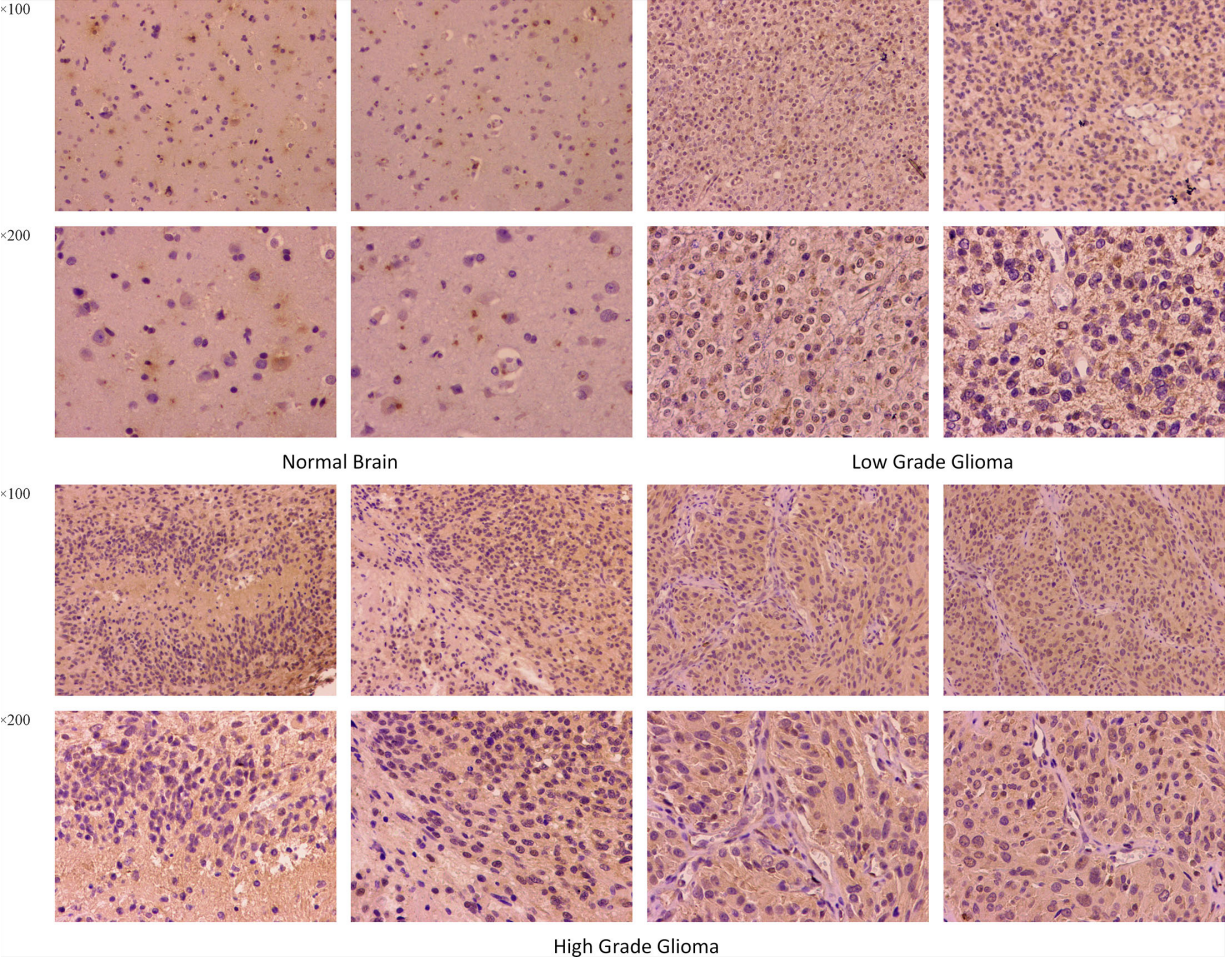
Immumohistochemical staining for TNFRSF12A expression in normal brain, low-grade glioma and high-grade glioma tissues. Magnification, ×100, ×200.

The differences in TNFRSF12A expression in normal brain, low-grade glioma, and high-grade glioma tissues were detected using immunohistochemical staining. The results revealed that TNFRSF12A was primarily expressed in the cytoplasm of cells, and TNFRSF12A expression was significantly higher in gliomas compared with normal brain tissue **(** **Figure 12** **)**. In addition, the expression of TNFRSF12A was significantly higher in high-grade gliomas than low-grade gliomas **(** **Figure 12** **)**. The immunohistochemical staining results validated the previous dataset analyses indicating that TNFRSF12A expression was progressively up-regulated in normal tissues, low-grade gliomas, and high-grade gliomas.

## Discussion

Currently, 19 tumor necrosis factors (TNF) and 29 tumor necrosis factor receptors (TNFR) have been identified in humans. After binding, these receptors and ligands maintain the body’s homeostasis by regulating cytokine production and controlling cell survival. Numerous studies have shown that these proteins function in human immune responses (11–13). Numerous TNF family proteins are highly expressed in tumors and exert modulatory effects (14). For example, tumor necrosis factor receptor 12 (TNFR12) regulates the immune tolerance of B cells and myeloid-derived suppressor cells (15) while tumor necrosis factor receptor 14 (TNFR14) regulates the immune activation of T cells (16).

Using a comprehensive analysis based on multiple databases, we focused on TNFRSF12A, which is significantly overexpressed in gliomas (17). TNFRSF12A, member 12A of the tumor necrosis factor receptor superfamily, also known as fibroblast growth factor-inducible 14 (FN14), is widely expressed in most healthy tissues but exhibits low expression in the brain (18, 19). Tumor necrosis factor-like weak inducer of apoptosis (TWEAK), the ligand of TNFRSF12A, is a type II transmembrane protein (20). Together, they constitute the TWEAK/TNFRSF12A signaling pathway that is involved in multiple biological processes, including proliferation, differentiation, migration, cell death (apoptosis and necrosis), angiogenesis, and inflammation (21, 22). The expression of TNFRSF12A markedly increases in damaged tissue, autoimmune diseases, and inflammatory diseases such as SLE and autoimmune myocarditis (23, 24). Numerous studies have shown that TNFRSF12A participates in the regulation of essential signaling pathways in many tumors. In gastric cancer, TNFRSF12A is involved in the activation of the PI3K/Akt and NF-kB signaling pathways, which ultimately leads to the development of gastric cancer (25, 26). TNFRSF12A also participates in activating the JAK/STAT signaling pathway in non-small cell lung cancers (27) and the NF-kB signaling pathway in prostate cancer (28, 29). The signaling pathways activated by TNFRSF12A ultimately lead to tumor cell invasion and migration. TNFRSF12A was found to be significantly overexpressed in gliomas. The overexpression of TNFRSF12A in glioma cell lines significantly increased cell migration and invasion, which demonstrated the tumor-promoting effects of TNFRSF12A (17, 30). It has been reported that the expression of TNFRSF12A was significantly higher in recurring gliomas than in newly diagnosed primary tumors (31). In our study, we found that the TNFRSF12A expression levels increased with the grade of the glioma. This observation is supported by several previous studies (17, 32). Thus, we suggest that TNFRSF12A contributes to the progression of glioma. According to survival analysis, TNFRSF12A expression was associated with a shortened survival in glioma patients. Previous studies have demonstrated that overexpression of TNFRSF12A in tumors results in malignancy (17, 33). However, these experimental interventions might present stronger specific effects than the actual physiological processes in the tumor because the experimental process might result in TNFRSF12A expression levels that far exceed those in actual tumors (34).

Anti-TNFRSF12A antibodies can inhibit tumor growth moderately and significantly prolong life expectancy by alleviating tumor-induced weight loss (35). This suggests that anti-TNFRSF12A antibodies prevent tumors from auto-damage and deterioration, which could preserve body mass. Interestingly, TNFRSF12A presented high integrity and independence with respect to its signaling processes and regulated downstream pathways without modification (36). Therefore, our results supported the conclusion that TNFRSF12A expression could serve as an independent high-risk predictor for glioma patients.

Currently, the administration of temozolomide (TMZ) can prolong the survival of a subset of glioma patients to some extent. However, most patients develop therapeutic resistance during treatment (37). With the accumulation of oncogenetic mutations, low-grade gliomas eventually are likely to develop into high-grade gliomas (38). Specifically, TMZ treatment results in genetic alterations and biological changes in GBM cells. For example, mutations occurring at high frequencies can result in DNA mismatch repair (39). Moreover, the accumulation of mutations also causes over-activation of the PI3K/Akt/mTOR signaling pathway (40, 41). TNFRSF12A is expressed at low levels in TMZ-sensitive gliomas and highly expressed in TMZ-resistant gliomas. Moreover, in TMZ-sensitive and TMZ-resistant glioma cell lines, lower and higher TNFRSF12A levels were expressed, respectively. Cells with drug-resistant properties exhibited an enhanced migratory capacity compared to cells without drug-resistant properties. This suggests that TNFRSF12A might be responsible for the increased migration of drug-resistant tumor cells (42). Glioma cells that are less sensitive to TMZ presented higher expression of TWEAK, TNFRSF12A, and NF-kb. Thus, the TWEAK/TNFRSF12A/NF-kb axis might participate in the drug resistance exhibited by some gliomas (43).

IDH was chosen as a classification criterion by WHO in 2016. Patients with IDH mutations presented significantly longer survival periods than those without mutations (44, 45). Furthermore, IDH mutations increased sensitivity to TMZ by disrupting the repair process of parp1-mediated DNA (46). In our analysis, TNFRSF12A was more highly expressed in IDH wild-type gliomas than gliomas with IDH mutations. One study reported that TNFRSF12A promoted the invasive phenotype of IDH1 wild-type gliomas, while IDH1-mutant gliomas exhibited low TNFRSF12A mRNA and protein levels compared with IDH1 wild-type gliomas (47).

Modern oncology has begun to experiment with gene target therapy, which is characterized by the fact that target drugs can focus on individual genes or proteins and affect specific cell types of tumors without many of the side effects associated with traditional chemotherapeutic drugs (48). There are numerous potential approaches to tumor therapy using TNFRSF12A as a target. With the goal of inhibiting the TWEAK/TNFRSF12A signaling pathway, Yin et al. studied a preparation called RG7212, which inhibits TWEAK binding to TNFRSF12A. RG7212 effectively inhibited tumor growth in athymic (nude) mice tumor xenograft models of renal cell carcinoma (ACHN, Caki-1), breast cancer (MDA-MB-231), and non-small cell lung cancer (Calu-3) (49). Roos et al. identified aurintricarboxylic acid (ATA) as an inhibitor of TWEAK/TNFRSF12A/NF-κB signaling. Through inhibition of Rac1 activation, ATA inhibited the TWEAK-induced glioma cell invasion process but did not affect cell viability or TNFRSF12A expression (50). It has been well-established that excessive activation of the TWEAK/TNFRSF12A signaling pathway promotes glioma growth (17, 33). However, it has also been reported that the function of this pathway can be achieved by the expression of TNRSF12A alone (36). Therefore, it is not certain whether inhibition of this signaling pathway could provide possible clinical therapeutic effects in glioma treatment.

Preparations made by combining a targeted polypeptide with a toxin is termed a targeted toxin, which is a class of drugs that can be internalized by and kill tumor cells (51, 52). Currently, the anti-TNFRSF12A monoclonal antibody, ITEM4, has been used for this purpose (53–55). Researchers have conjugated ITEM4 with recombinant gelonin (rGel), and this preparation exhibits significant anticancer properties in bladder cancer cell xenografts (53). In another study that used TNFRSF12A as the target, researchers synthesized an immunoconjugate using recombinant gelonin toxin and ITEM4, which produced significant tumor-inhibiting results in a breast cancer xenograft model (54). Zhou et al. used ITEM4 as an antibody to study two immunotoxins. One immunotoxin was a chemical conjugate composed of the rGel toxin and the anti-TNFRSF12A antibody, ITEM-4, and the other was a humanized, dimeric single-chain antibody of ITEM-4 fused to rGel. Both immunotoxins produced significant inhibitory effects on melanoma (MDA-MB-435) in xenograft mice (55). Importantly, these studies involved *in vivo* experiments, demonstrating that TNFRSF12A is a viable potential immune target.

To achieve more effective drug delivery, researchers have developed novel nanomaterial methodologies. They have processed synthetic nanomaterials with a 100 nm carboxylate-modified polystyrene modification combined with ITEM4. This new mode of administration has many advantages. First, it selectively binds TNFRSF12A, but not the brain extracellular matrix, which reduces the non-specific binding of targeted nanoparticles in the brain. Second, it can associate with and be internalized by TNFRSF12A-positive GBM cells. Finally, it has good tissue penetration. A previous study demonstrated that nanoparticles targeting TNFRSF12A more accurately localized to gliomas compared to untargeted TNFRSF12A nanoparticles (56). Recently, Wadajkar et al. synthesized degradable nanoparticles by processing poly(lactic-co-glycolic acid) (PLGA) and PLGA-polyethylene glycol (PLGA-PEG) polymers. Nanoparticles bound to ITEM-4 exhibited minimal binding to extracellular brain components, extremely strong binding to TNFRSF12A, and increased uptake into brain tumor cells. Compared with unbound ITEM-4 nanoparticles, ITEM-4-bound nanoparticles were retained longer in the tumor (57). In summary, multiple research results have proven that TNFRSF12A is a potential glioma therapeutic target.

## Conclusion

Overall, TNFRSF12A is significantly overexpressed in gliomas and closely associated with inflammatory processes. Studies have revealed that specific drug modifications can improve the precision therapy of TNFRSF12A for gliomas. Our analysis provides a more comprehensive demonstration of the roles of TNFRSF12A in glioma progression. In conclusion, TNFRSF12A can serve as an independent risk factor to predict prognosis and has tremendous value in glioma immunotherapy.

## Data Availability Statement

The original contributions presented in the study are included in the article/supplementary material. Further inquiries can be directed to the corresponding authors.

## Ethics Statement

The studies involving human participants were reviewed and approved by the Ethics Committee of the First Hospital of Shanxi Medical University. The patients/participants provided their written informed consent to participate in this study.

## Author Contributions

YZ, XY, and X-LZ contributed to the entire project, from the design proposal, to the collection and collation of data, to the writing of the paper. Z-ZW helped retrieve and organize the data, while HB and J-JZ were responsible for statistical analysis. C-YH and H-BD are responsible for supervising and providing financial support. All authors contributed to the article and approved the submitted version.

## Funding

This work was supported by the National Natural Science Foundation of China Youth Fund (30600637); China Postdoctoral Science Foundation Special Grant (2019T120195); Key Grants for Returning Students from Shanxi Province (2016-4).

## Conflict of Interest

The authors declare that the research was conducted in the absence of any commercial or financial relationships that could be construed as a potential conflict of interest. 

## Abbreviations

LGG, low-grade glioma; GO, Gene Ontology; KEGG, Kyoto Encyclopedia of Genes and Genomes; PPI, Protein-Protein Interaction; IHC, immunohistochemistry.
